# Amino Acid Substitutions HA A150V, PA A343T, and PB2 E627K Increase the Virulence of H5N6 Influenza Virus in Mice

**DOI:** 10.3389/fmicb.2018.00453

**Published:** 2018-03-13

**Authors:** Xiuming Peng, Fumin Liu, Haibo Wu, Xiaorong Peng, Yufan Xu, Liyan Wang, Bin Chen, Tao Sun, Fan Yang, Shujing Ji, Nanping Wu

**Affiliations:** Collaborative Innovation Center for Diagnosis and Treatment of Infectious Diseases, State Key Laboratory for Diagnosis and Treatment of Infectious Diseases, The First Affiliated Hospital, School of Medicine, Zhejiang University, Hangzhou, China

**Keywords:** avian influenza virus, H5N6, virulence, adaption, mice, amino acids

## Abstract

H5N6 avian influenza viruses (AIVs) can cause severe pneumonia and death in humans. However, the molecular determinants of H5N6 influenza virus mammalian adaption are still unclear. Three amino acid substitutions (HA A150V, PA A343T, PB2 E627K) are observed in H5N6 virus A/duck/Zhejiang/6D2/2013 (6D2) in lung-to-lung passage in mice. These substitutions are crucial to the pathogenicity of mouse-adapted virus. In this study, we investigated the contribution of each amino acid substitution in the virus by reverse genetics. The results demonstrate that HA A150V greatly altered the receptor binding preference of 6D2. Virus bearing this substitution acquired increased mortality than mice infected with wild-type 6D2. The PA A343T substitution mildly enhanced viral polymerase activity but the reduced survival rate in mice indicates this substitution may change the immunoreaction of the host. The well-known PB2 E627K substitution increased eight folds the relative polymerase activity compared to PA A343T and resulted in 100% death rate in mice. In addition, we show that PA A343T dramatically exacerbates the effect of PB2 E627K on viral polymerase activity; when combined, these two substitutions work synergistically. However, HA A150V and PA A343T seemed to attenuate PB2 E627K *in vivo*, which implies the difference between mixed viral populations under natural condition and single population under experiment, specialization and cooperation in quasispecies is important in the process of adaption. This study suggests that HA A150V, PA A343T, and PB2 E627K are crucial in the adaption and increased pathogenicity of H5N6 in mammalian hosts.

## Introduction

H5 subtype avian influenza viruses (AIVs) can transmit directly to humans from wild or domestic birds and causes high mortality, raising concerns of potential pandemic threats. Since 2014, sporadic human cases of H5N6 have been documented. As of September 2017, WHO reported 16 laboratory-confirmed cases of human infection with H5N6 AIVs in China; 11 of the 16 cases were fatal, bringing the case-fatality rate to 69% (WHO, 2017). Recent studies suggest an increase of human like receptor binding affinity in avian H5N6 in China (Bi et al., 2016; Sun et al., 2016). Avian-to-human transmission is now a significant threat to public health.

Avian influenza viruses frequently infect across species and cause pandemics in mammals (Gilsdorf et al., 2006; Zhang et al., 2013; Yu et al., 2014). There are two crucial steps in the cell infection process of influenza virus, entry and replication (Hu and Liu, 2015). Consequently, among the amino acid substitutions documented in the process of AIVs’ adaption to other hosts, substitutions in hemagglutinin (HA) and viral polymerase carry relatively more importance (Ping et al., 2010; Linster et al., 2014; Yang et al., 2017; Zhang et al., 2017).

Hemagglutinins in the influenza virus binds receptors on host cells, closely related to host range (de Vries et al., 2011; Teng et al., 2016; Tzarum et al., 2017) and are involved in pathogenicity (Zhao et al., 2017). The HAs from AIVs preferentially bind to α2,3-linked sialic acid (SAα2,3) receptors, however, human influenza viruses have a higher affinity to SAα2,6 receptors (Rogers and Paulson, 1983; Matrosovich et al., 2000; Glaser et al., 2005). Receptor binding preference is a crucial component in adaption, pathogenicity, and transmission (citation updated and moved to backward), with only a few substitutions, for instance, HA D190E, D225G, Q192H, and K193R (Glaser et al., 2005; Stevens et al., 2006, 2008; Watanabe et al., 2011), influenza virus can change receptor specificity, from avian type to human type.

The transcriptional activity of avian influenza viral polymerase complex in human cells is restricted (Manz et al., 2013), consequently, amino acid substitutions in viral polymerase complex are also important in adaption and virulence. For example, polymerase acidic (PA) R443 K, PA-K356R, polymerase basic 2 protein (PB2) E627K, PB2 D701N, and polymerase basic protein 1 (PB1) D622G) are all associated with replication and host tropism (Hatta et al., 2001; Czudai-Matwich et al., 2014; Feng et al., 2015; Zhao et al., 2015; Xu G. et al., 2016).

Amino acid substitutions are associated with *trans*-species adaptation, including transmission and replication. However, the molecular mechanism underlying the highly virulent H5N6 in humans is still unclear. Recently, we reported that a mouse-adapted (MA) H5N6 influenza virus A/duck/Zhejiang/6D2/2013 (6D2) carried several amino acid substitutions, including HA A150V, PA A343T, and PB2 E627K in the process of mouse lung-to-lung passage (Peng et al., 2016). In this study, we used reverse genetics to verify the role of these amino acid substitutions in mouse adaption.

## Materials and Methods

### Ethics Statement and Risk Assessment

All applicable international, national, and/or institutional guidelines for the care and use of animals were followed. All the animal experiments performed were approved by The First Affiliated Hospital, School of Medicine, Zhejiang University (No. 2015-15). All experiments using H5N6 AIVs were performed in a biosafety level 3 laboratory.

### Cells

Human embryonic kidney (293T) cells, Madin-Darby canine kidney (MDCK) cells, and adenocarcinomic human alveolar basal epithelial (A549) cells were propagated and maintained in Dulbecco’s modified Eagle’s medium (DMEM, Gibco, United States) supplemented with 10% fetal bovine serum (Gibco, United States), non-essential amino acids (Gibco, United States), and antibiotics (Gibco, United States). Cells were used for virus propagation, plasmid transfection, and tissue culture infectious doses (TCID50) determination.

### Molecular Cloning and Generation of Recombinant Viruses

Plasmid-based reverse genetics was performed as described previously (Hoffmann et al., 2000), with minor modifications. Briefly, the cDNA of the virus was synthesized by viral RNA using primer Uni-12 (Hoffmann et al., 2001) and PrimeScript^TM^ RT reagent Kit (TAKARA, Japan), then amplified by PCR using 6D2 gene specific primers and PrimeSTAR Max DNA Polymerase (TAKARA, Japan). This was then cloned into pHW2000 plasmid (Hoffmann et al., 2000). Single point mutations were introduced using a previously described method (Liu and Naismith, 2008). Eight plasmids were cotransfected to 293T cells using Lipofectamine 2000 Reagent (Invitrogen, United States). The culture supernatant was then harvested 2 days post transfection and embryonated eggs were infected and incubated for 2 days (Song et al., 2009; Tan et al., 2014). The allantoic fluid was aliquoted and stored in -80°C.

### Receptor Binding Assay

We used hemagglutinating receptor-specific red blood cells (RBCs) to determine receptor binding specificity using a previously described method (Liu et al., 2014; Jin et al., 2016). Briefly, normal chicken RBCs (contain SAα2,3 and SAα2,6 receptors) and sheep RBCs (contain largely SAα2,3 receptors) were washed three times with phosphate buffered saline (PBS, HyClone, United States). The chicken RBCs were incubated with α2,3 neuraminidase (NEB, United States) for 1 h and then washed with PBS three times (to generate RBCs with only SAα2,6 receptors) according to the manufacturer’s instruction. RBCs were resuspended in PBS to obtain 0.5% RBCs. A/pigeon/Zhejiang/727097/2014 H5N1 and A/Puerto Rico/8/1934 H1N1 were used as controls. Fifty microliters of twofold serially diluted virus was incubated with 50 μL of 0.5% RBCs in U bottom microtiter plates for 1 h. The highest RBCs hemagglutination titer was recorded.

### Minigenome Assay for Determining Polymerase Complex Activity

The minigenome assay was performed using a system described previously (Song et al., 2014; Tan et al., 2014), with minor modifications. Briefly, 293T cells were seeded and cultured in a 24-well plate overnight, transfected with six reconstructed plasmids: 100 ng of pHW2000-NP, pHW2000-PA (wild-type 343A or mutated 343T), pHW2000-PB1, pHW2000-PB2 (wild-type 627E or mutated 627K), pFluc (a firefly luciferase reporter plasmid containing a noncoding sequence from the NP segment of influenza A virus), and 20 ng of pRL-TK (expresses Renilla luciferase, Promega, United States) per well. Forty-eight hours following transfection, cell lysates were prepared and luciferase yield was measured using the Dual-Luciferase Reporter Assay System (Promega, United States). The polymerase activity was calculated by normalizing firefly luciferase activity to Renilla luciferase activity. The experiments were conducted in independent triplicate wells.

### Virus Replication Assay in Cells

Triplicate A549 cells and MDCK cells (10^5^ cells per well) were infected with different viruses at a multiplicity of infection (MOI) of 0.01 and incubated at 37°C for 1 h. Cells were then washed with PBS and further incubated with culture medium (supplemented with 2 μg/ml TPCK-Trypsin [Worthington, United States]). Supernatants were collected at 24, 48, and 72 h and subjected to virus tittering by TCID50. Virus titer was determined by end point titration in MDCK in 96-well plates (de Wit et al., 2004). Briefly, 10-fold serial dilution were conducted and 100 μl/well of virus was added to confluent MDCK cells, plates were then incubated at 37°C, 5% CO_2_ for 72 h. Each sample consisted of four replicates, and TCID50 was analyzed in using hemagglutinating of chicken RBCs, following the Reed and Muench method (Reed and Muench, 1938).

### Mouse Pathogenicity Experiments

*In vivo* experiments were performed using the methods of previous study (Song et al., 2009; Bussey et al., 2010), with a few adjustments. For the measurements of virus titer in lungs of mice, six groups (six mice per group) of 6-week-old BALB/c mice were anesthetized by isoflurane and inoculated intranasally with 10^6^ TCID50 virus in 50 μL allantoic fluid. Three mice in each group were euthanized at day 3 and day 6 post inoculation. Lungs of mice were collected, homogenized, and resuspended in PBS. The supernatants were used for virus titration using TCID50. The data is represented as the mean of TCID50 ± standard deviation. Mice (five mice per group) were infected with 10^6^ TCID50 virus in 50 μL PBS and observed for weight loss and mortality for 14 days post infection. Lungs from infected mice were fixed in 10% neutral buffered formalin for 3 days, embedded in paraffin, cut to 4 μm-sections, then subjected to hematoxylin–eosin staining (H&E staining).

### Statistical Analysis

A one-way ANOVA was used to determine statistical significant between experimental groups. *P* < 0.05 is considered as statically significant.

## Results

### Generation of Reassortant Viruses

**FIGURE 1:**
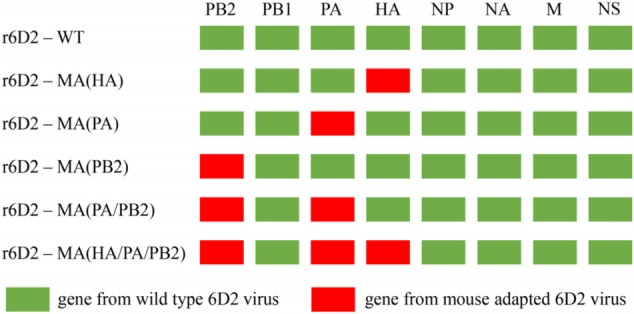
Schematic of reassortant viruses with different plasmid combinations.

Three amino acid substitutions (HA A150V, PA A343T, and PB2 E627K) discovered in a previous study were examined (Peng et al., 2016). A reverse genetic system was used to study the function of the three substitutions. The rescued six 6D2, wild-type (WT) or mouse-adapted viruses, bearing different combinations of the three amino acid substitutions: no amino acid substitution, HA, PA, PB2, and PA/PB2, or all three substitutions are designated as r6D2-WT, r6D2-MA (HA), r6D2-MA (PA), r6D2-MA (PB2), r6D2-MA (PA/PB2), r6D2-MA (HA/PA/PB2), respectively (**Figure 1**).

### All Three Amino Acid Substitutions Increase the Virulence of 6D2 in Mice

**FIGURE 2:**
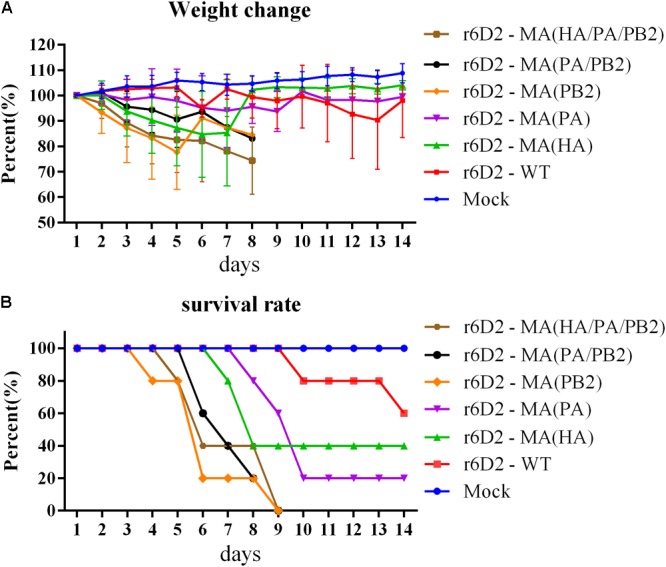
Weight change and survival rate of mice inoculated with 6D2 virus. **(A)** The weight change of mice inoculated with 10^6^ TCID50 virus in 50 μl. **(B)** The survival rate of mice inoculated with 10^6^ TCID50 virus in 50 μl.

Six groups of BALB/c mice were infected and the weight change and survival rate of mice was recorded (**Figures 2A,B**). The r6D2-MA (HA/PA/PB2) and r6D2-MA (PB2) group suffered the most drastic weight loss (average weight losses were 17% and 22.5% at day 5, respectively, **Figure 2A**). The weight of the r6D2-MA (HA) group declined sharply (average weight loss was 12.7% at day 5), indicating that this substitution plays a role in the increase of virulence. All the mice in group r6D2-MA (PB2), r6D2-MA (PA/PB2), and r6D2-MA (HA/PA/PB2) died prior to day 9 post infection. The 100% death rate demonstrates the pivotal role of PB2 E627K in H5N6. HA A150V alone caused a death rate of 60% (**Figure 2B**). PA A343T alone decreased the survival rate of mice compared with wild-type 6D2 (**Figure 2B**). However, HA A150V or PA A343T seems to attenuate PB2 E627K *in vivo* (**Figure 2B**).

At day 3 post infection, the average lung virus titer of r6D2-MA (HA) and r6D2-MA (PA) is lower than r6D2-WT (*P* > 0.05), implying that the high death rate caused by these two substitutions may not due to high virus titer. All virus containing PB2 E627K grew faster, yielding more than 1,000 folds virus titer than the wild-type virus (**Table 1**).

**Table 1:** The virus titer in lungs of BALB/c mice infected with 6D2 influenza virus.

	Average titer (mean log_10_ TCID50 ± SD)	
Virus	Day 3	Day 6
r6D2-WT	1.22 ± 2.12	3.17 ± 2.75
r6D2-MA (HA)	0.79 ± 1.37	2.65 ± 1.06
r6D2-MA (PA)	0.83 ± 1.44	0.44 ± 0.77
r6D2-MA (PB2)	5.22 ± 1.17	4.56 ± 1.07
r6D2-MA (PA/PB2)	5.22 ± 0.96	3.08 ± 0.83^a^
r6D2-MA (HA/PA/PB2)	8.07 ± 0.54^∗∗^	3.89 ± 0.59

*Three BALB/c mice were inoculated with 10^*6*^TCID50 virus in 50 μL. Three mice in each group were euthanized for lung virus titration on day 3 and day 6 post inoculation. Lungs of mice were collected, homogenized, and the supernatants were used for virus titration using TCID50 in MDCK cells. The titration of virus is presented with mean log_*10*_ TCID50 ± SD. Statistical difference was measured with one-way ANOVA, followed with a Bonferroni correction, ^∗∗^P < 0.01 [r6D2-MA (HA/PA/PB2) vs. r6D2-WT],^*a*^ one of the three mice died.*

**FIGURE 3:**
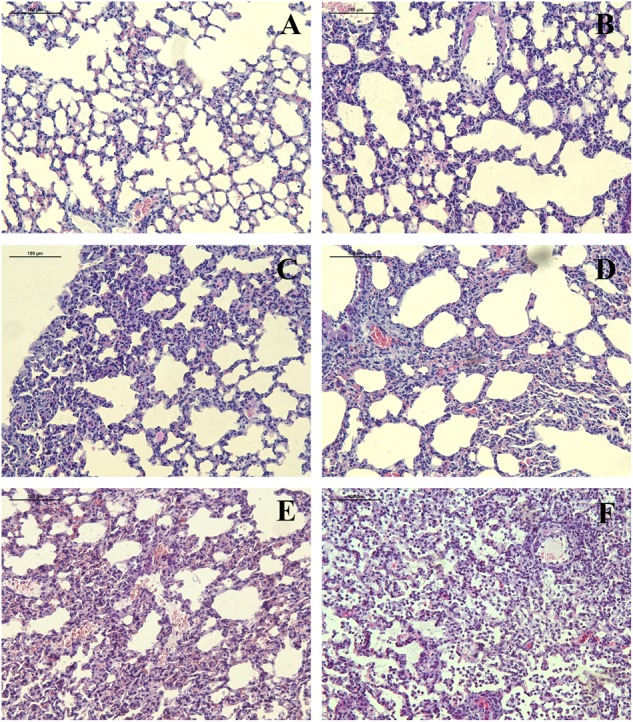
Pathological changes of lung tissue from 6D2 infected mice. **(A–F)** Lung tissue from mice infected by r6D2-WT, r6D2-MA (HA), r6D2-MA (PA), r6D2-MA (PB2), r6D2-MA (PA/PB2), and r6D2-MA (HA/PA/PB2), respectively. Lung sections were subjected to hematoxylin–eosin staining and displayed at a magnification of ×200.

The H&E staining of mouse lungs infected with r6D2-WT is morphologically normal (**Figure 3A**). r6D2-MA (HA) and r6D2-MA (PA)-inoculated mice showed thickening of the alveolar wall (**Figures 3B,C**). The mice infected with recombinant r6D2-MA (PB2) and r6D2-MA (PA/PB2) displayed severe inflammatory cell infiltration, hemorrhage, and bronchopneumonitis (**Figures 3D,E**). The pathological changes of r6D2-MA (HA/PA/PB2) infected mice are the most severe (**Figure 3F**).

### Growth Kinetic of Reassortant Viruses in A549 and MDCK Cells

**FIGURE 4:**
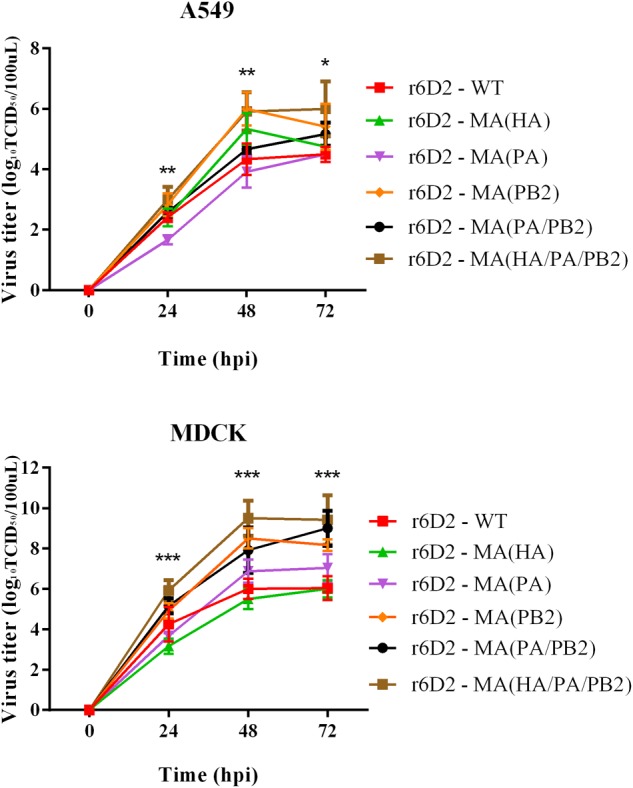
Virus titer in supernatant of A549 and MDCK following infection. Triplicate cells (10^5^ cells per well) were infected with different viruses at a multiplicity of infection (MOI) of 0.01. Supernatants were collected at 24, 48, and 72 h and subjected to virus tittering by TCID50. Three independent experiments were presented per point with mean value and standard deviation (SD), ^∗^*P* < 0.05, ^∗∗^*P* < 0.01, ^∗∗∗^*P* < 0.001, one-way ANOVA (Bonferroni correction adopted, see Supplementary Tables S1, S2).

To explore the contribution of mouse adapted substitutions to growth ability in mammalian cells, we used six reassortant viruses to infect A549 and MDCK cells (**Figure 4**). In MDCK cells, the titer of virus containing PA A343T is ten times the wild-type virus (*P* > 0.05), but not in A549. This suggests that PA A343T alone is not sufficient to induce strong *in vitro* replication. The virus titer of r6D2-MA (PB2) and r6D2-MA (HA/PA/PB2) grew similarly (viral titers were both 10^6^ TCID50/100 μl) in A549 at 48 h post infection, 50 times higher than r6D2-WT (*P* < 0.01), indicating that PB2 E627K increased replication efficiency markedly. r6D2-MA (HA) grew more efficiently than r6D2-WT in A549 cells at 48 h post infection (virus titers were 10^5.3^ and 10^4.3^ TCID50/100 μl, respectively, *P* < 0.05), demonstrating that HA A150V is responsible for the enhanced growth in human cells.

### HA A150V Plays a Key Role in Mammalian Receptor Affinity

**FIGURE 5:**
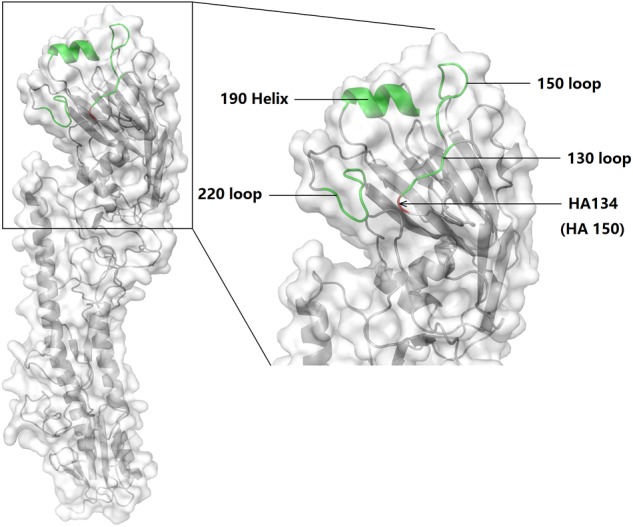
Schematic of the HA subunit and receptor binding site of 6D2. The 130 loop, 150 loop, 220 loop, and 190 helix were labeled. HA 150 [in another study (Auewarakul et al., 2007), this point is referred to as HA134] is indicated with an arrow. The molecular model was built using PyMol (v1.8.0.0; Schrödinger, LLC).

To determine the mechanism of HA A150V in the increased mouse death rate and virus titer in A549, we conducted homologous molecular modeling (**Figure 5**) using the Swiss-model (Arnold et al., 2006; Bordoli et al., 2009; Biasini et al., 2014). HA A150V localizes near the 130 loop of the receptor domain (Skehel and Wiley, 2000), probably influencing receptor binding. HA protein of influenza binds the cell surface receptors with SA-linked glycoproteins. Avian influenza virus preferentially binds to receptors with SAα2,3 receptors, while human influenza virus preferentially binds the SAα2,6 receptors (Rogers and Paulson, 1983; Matrosovich et al., 2000; Glaser et al., 2005).

The hemagglutination assay suggests that HA A150V completely abolishes the binding of HA with SAα2,3 receptors, but enhanced binding with SAα2,6 receptors (**Table 2**). To test whether the substitution found in mouse adaption is also relevant in human strains, we examined all the sequences of H5 influenza A virus in the Influenza Virus Database of NCBI (Bao et al., 2008). Interestingly, we found 11 strains (8,008 strains of H5 influenza in total) with valine in HA 150 in the database as of November 2017. Of those, 91% (10/11) of the viruses are clinical human strains (**Table 3**).

**Table 2:** Hemagglutination titer of virus in three kinds of RBCs containing different types of receptors.

		Receptor binding specificity		
Virus	HA 150	α-2,3	α-2,3 and α-2,6	α-2,6
A/pigeon/Zhejiang/727097/2014 H5N1	–	256	256	<2
A/Puerto Rico/8/1934 H1N1	–	<2	128	256
rg6D2-WT	A	512	128	256
rg6D2-MA (HA)	V	<2	256	1024
rg6D2-MA (HA/PA/PB2)	V	<2	512	1024

*Hemagglutination titer of virus in three kinds of RBCs containing only SAα2,6 receptors, SAα2,6 receptors and SAα2,3 receptors, and only SAα2,3 receptors, respectively. Hemagglutination titers were calculated using the highest virus dilution.*

**Table 3:** All H5 viruses with valine (V) in HA150 from Influenza Virus Database of NCBI.

Virus strain	Accession	Host	HA aa at position 150
A/Anhui/1/2006 (H5N1)	CY098668	Human	V
A/Cambodia/V0401301/2011 (H5N1)	JN588807	Human	V
A/Egypt/N01644/2010 (H5N1)	CY062470	Human	V
A/Hanoi/03/2004 (H5N1)	AJ715872	Human	V
A/Hubei/1/2006 (H5N1)	CY098641	Human	V
A/Jiangxi/1/2005 (H5N1)	FJ492885	Human	V
A/Thailand/676/2005 (H5N1)	DQ360835	Human	V
A/Viet Nam/3046/2004 (H5N1)	AY651335	Human	V
A/Vietnam/CL105/2005 (H5N1)	DQ497726	Human	V
A/Vietnam/UT31312II/2007 (H5N1)	HM114577	Human	V
A/duck/Vietnam/NA72/2007 (H5N1)	JX021305	duck	V

*This table summarizes all H5 viruses with valine (V) in HA150 from the NCBI Influenza Virus Database. aa, amino acid.*

### The Amino Acid in PA 343 and PB2 627 Influence the Polymerase Activity of 6D2

**FIGURE 6:**
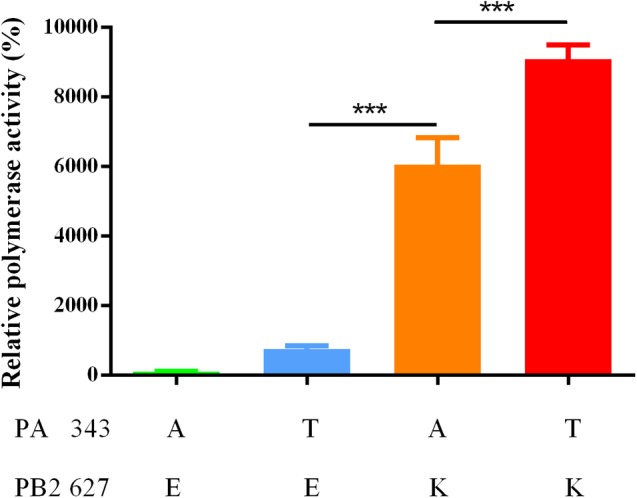
Luciferase activity of complexes with different combination of mutants. The polymerase activity was calculated by normalizing firefly luciferase activity to Renilla luciferase activity. The data is shown by the mean of relative polymerase activity with standard deviation (SD) of triplicate wells. ^∗∗∗^*P* < 0.001, one-way ANOVA.

The PA and PB2 proteins are components of viral RNA polymerase. We hypothesized that PA A343T and PB2 E627K affect the polymerase activity of reconstituted ribonucleoprotein (RNP) complexes (**Figure 6**). 293T cells were transfected with reconstructed plasmid expressing wild-type and mutated NP, PA, PB1, and PB2. The polymerase activity of the RNP containing PA 343T is 22 folds higher than wild-type (*P* > 0.05). Therefore, PA A343T alone is not sufficient to enhance replication ability substantially. Notably, the polymerase activity of the RNP containing PB2 627K alone or combined PA 343T and PB2 627K are 182 and 273 folds higher than wild-type (*P* < 0.001), respectively. Combining the two substitutions has a synergistic effect on polymerase activity.

## Discussion

H5N6 influenza virus continues to circulate in poultry in Asia and the Pacific region (OIE World Organisation for Animal Health, 2016). Some mammal (Cao et al., 2017; Kim et al., 2017) and human cases are also reported occasionally (Yang et al., 2015; Pan et al., 2016; Xu W. et al., 2016). To investigate the mammal adaption mechanism of H5N6, mouse adaption was conducted (Peng et al., 2016). Our results suggest that amino acid substitutions in HA and viral polymerase contributed to viral growth in mice. We further explored the potential mechanism and concluded that HA A150V altered receptor binding preference, from avian to human type. PA A343T and PB2 E627K improved the efficiency of viral polymerase. All three substitutions contributed to the adaption of 6D2 AIV.

The homology modeling result (**Figure 5**) indicates that HA 150 localizes near the 130 loop of the receptor binding site. We speculate this substitution is related with enhancement of receptor binding affinity. Crystallographic studies show that when compared with 150A (position 134 in their study), 150V increased the distance of 130 loop and 220 loop in the receptor-binding site (Crusat et al., 2013). Crusat et al. (2013) used the HA from H5N1 and the other seven segments from PR8 and concluded that HA A150V reduced the affinity to avian like receptor with no human like receptor binding increase detected. However, our receptor binding assay suggests the recombinant H5N6 virus bearing HA A150V not only abolished the SAα-2,3Gal affinity, but also greatly enhanced SAα-2,6Gal binding. Using solid-phase direct binding assay, another study indicates that the HA A150V stabilized the interaction between SAα2,6Gal and the virus receptor binding site. However, they didn’t generate the virus containing only HA A150V (Auewarakul et al., 2007). Our study revealed the role of HA A150V in the binding of human like receptor, as shown by virulence enhancement in mice and increase of virus titer in A549 cells.

The C-terminal domain of PA contains the PB1 binding site; PA residues 239–716 have been shown to interact with PB1 (Obayashi et al., 2008). PA A343T may affect the binding between these two subunits and change the efficiency of viral polymerase. Another study indicated that PA A343T alone does not significantly enhance the replication capability (Yamaji et al., 2015). Although we didn’t observe any contribution by PA A343T to replication in A549 and MDCK (**Figure 4**), our results suggest that PA A343T mildly increases polymerase activity when compared to the wild-type influenza polymerase (**Figure 6**). This is not sufficient to explain the increased pathogenicity *in vivo* (**Figures 2, 3**). These results imply that pathogenicity is not only determined by virus growth but also by the immunoreaction to infection, including cytokine storm (Sakabe et al., 2013; Guo et al., 2015), and inflammasome-related innate immunity (Huang et al., 2013). The detailed mechanism for PA in altering the virulence is still not fully understood.

PB2 E627K is a well-characterized substitution in mammalian adaption of avian influenza viruses. According to the Influenza Virus Database of NCBI (Bao et al., 2008), five of nine available H5N6 influenza virus isolated from human (Bi et al., 2016) have mammalian type amino acid K in the position 627 in PB2. In our study, this substitution is also vital for the H5N6 mammalian adaption. However, with a single PB2 E627K, the avian influenza virus polymerase activity is still lower than the seasonal human influenza virus (Arai et al., 2016). This suggests that the avian influenza virus requires additional substitutions to proliferate in human cells. The finding that combined PA A343T and PB2 E627K act synergistically in minigenome assay provides a new avenue for influenza virus adaption research.

All viruses containing single substitution has a lower mice survival rate than r6D2-WT, however, the results of cell infection and *in vivo* study seems that virus containing HA A150V and/or PA A343T attenuate PB2 mutation (**Figure 2B**). RNA-dependent RNA polymerase in influenza lacks proof-reading mechanism, in natural conditions, the form of existence can be a cloud of diverse variants called quasispecies *in vivo* (Domingo and Holland, 1997; Lauring and Andino, 2010). The substitutions are chosen according to the differences of sanger sequencing between the wild-type virus and mouse adapted virus (Peng et al., 2016), therefore, they don’t necessarily appear in a single viral particle, they possibly exist in different virions, in a specific pattern. Consensus sequence is still unable to explain the whole adaption process of the influenza virulence *in vivo*. Mixed viral populations grow better than single population (Xue et al., 2016), homogenous population tend to be less successful in the host environment (Lauring and Andino, 2010).

## Conclusion

This study revealed the significance of HA A150V, PA A343T, and PB2 E627K in the mammalian adaption process of an H5N6 avian influenza virus. The adaption of 6D2 consists of a change in receptor affinity from avian to human type, and the enhancement of polymerase activity. Our findings elucidate the mechanism of these substitutions in mammalian adaption and provides information on pathogenicity markers in the surveillance of avian influenza.

## Author Contributions

XmP and FL performed the research, analyzed the data, and drafted the paper. HW and NW designed the research and revised the manuscript. XrP, YX, LW, BC, TS, FY, and SJ performed the research. All authors read and approved the final version of the manuscript.

## Conflict of Interest Statement

The authors declare that the research was conducted in the absence of any commercial or financial relationships that could be construed as a potential conflict of interest.
