# Supplementary material for: Amino Acid Substitutions HA A150V, PA A343T, and PB2 E627K Increase the Virulence of H5N6 Influenza Virus in Mice
Source: Front Microbiol. 2018 Mar 13;9:453. doi: 10.3389/fmicb.2018.00453 (PMC5859062; doi:10.3389/fmicb.2018.00453)
Supplement: Supplementary file 1 [file Table_1.DOCX]

Table S1

| Cell type | Time | P value |
| --- | --- | --- |
| A549 | 24h | 0.002 |
|  | 48h | 0.001 |
|  | 72h* | 0.028 |
| MDCK | 24h | 0.000 |
|  | 48h | 0.000 |
|  | 72h | 0.000 |

Notes: This table shows the P value of ANOVA. *, although P<0.05, we didn’t find any significance in A549 at 72h when the groups compared in pairs (data not shown).
