# Supplementary material for: Amino Acid Substitutions HA A150V, PA A343T, and PB2 E627K Increase the Virulence of H5N6 Influenza Virus in Mice
Source: Front Microbiol. 2018 Mar 13;9:453. doi: 10.3389/fmicb.2018.00453 (PMC5859062; doi:10.3389/fmicb.2018.00453)
Supplement: Supplementary file 2 [file Table_2.DOCX]

Table S2

| Cell type | Time | Group vs group | P value |
| --- | --- | --- | --- |
| A549 | 24h | r6D2-MA (PA) vs r6D2-MA (PB2) | 0.004 |
|  |  | r6D2-MA (PA) vs r6D2-MA (PA/PB2) | 0.027 |
|  |  | r6D2-MA (PA) vs r6D2-MA (HA/PA/PB2) | 0.001 |
|  | 48h | r6D2-WT vs r6D2-MA (PB2) | 0.025 |
|  |  | r6D2-WT vs r6D2-MA (HA/PA/PB2) | 0.036 |
|  |  | r6D2-MA (PA) vs r6D2-MA (PB2) | 0.004 |
|  |  | r6D2-MA (PA) vs r6D2-MA (HA/PA/PB2) | 0.006 |
| MDCK | 24h | r6D2-WT vs r6D2-MA (HA/PA/PB2) | 0.021 |
|  |  | r6D2-MA (HA) vs r6D2-MA (PB2) | 0.014 |
|  |  | r6D2-MA (HA) vs r6D2-MA (PA/PB2) | 0.005 |
|  |  | r6D2-MA (HA) vs r6D2-MA (HA/PA/PB2) | 0.000 |
|  | 48h | r6D2-WT vs r6D2-MA (PB2) | 0.017 |
|  |  | r6D2-WT vs r6D2-MA (HA/PA/PB2) | 0.001 |
|  |  | r6D2-MA (HA) vs r6D2-MA (PB2) | 0.004 |
|  |  | r6D2-MA (HA) vs r6D2-MA (PA/PB2) | 0.021 |
|  |  | r6D2-MA (HA) vs r6D2-MA (HA/PA/PB2) | 0.000 |
|  |  | r6D2-MA (PA) vs r6D2-MA (HA/PA/PB2) | 0.011 |
|  | 72h | r6D2-WT vs r6D2-MA (PA/PB2) | 0.006 |
|  |  | r6D2-WT vs r6D2-MA (HA/PA/PB2) | 0.002 |
|  |  | r6D2-MA (HA) vs r6D2-MA (PA/PB2) | 0.005 |
|  |  | r6D2-MA (HA) vs r6D2-MA (HA/PA/PB2) | 0.002 |
|  |  | r6D2-MA (PA) vs r6D2-MA (HA/PA/PB2) | 0.032 |

Notes: This table shows the P value when P<0.05, Bonferroni correction is used in the multiple comparison.
